# Human Physiology

**Published:** 1835-07-01

**Authors:** 


*Human Physiology*, by John Elliotson, m.d. Cantab., f.r.s., &c. *With which is incorporated much of the Elementary Part of the Institutiones Physiologicæ of* J. F. Blumenbach, m.d., f.r.s., Professor in the University of Göttingen. *Illustrated with numerous Woodcuts. Fifth Edition.*—London, 1835. 8vo. Part I. pp. 302.

This, it appears, is the fifth edition of Dr. Elliotson's Physiology; but how it happens to be so, is somewhat enigmatical. The commentaries of the editor having gradually extended till they form the larger and more important part of the whole, the work may now fairly enough be entitled Elliotson's Physiology; but, seeing that, in the four preceding editions, it has been known to the world as Blumenbach's, we cannot perceive that the present is any thing more than the first edition of Elliotson's; and the only object in calling it the fifth appears to be, that the next may pass for the sixth: this is a very good way of gaining a march upon the public, but whether it be strictly consonant with literary honesty, we must leave to the moral philosophers to determine.

So much for bibliography, and now for physiology, of which the work before us, though destitute of any striking claims to originality, affords, on the whole, a learned and judicious compendium.

In reviewing systematic works of this kind, involving a great variety of subjects, we are obliged either to confine ourselves to a mere notice of their general merits, or to select a few topics for more particular discussion: we prefer the latter plan, because it affords us, from time to time, an opportunity of dilating on such points as seem to require further illustration,—of introducing matters that may have been altogether overlooked,—and, lastly, of putting the student on his guard against those gratuitous hypotheses, derived rather from the spirit of the time than the observation of nature, some one of which has been prevalent in every age, giving an impulse to science in a wrong direction; though not, perhaps, without some beneficial tendency, by enlisting the fancy, and even the passions, in the cause of philosophy, which might lose its interest, if reason alone were always engaged in it.

Our object, in short, is to contribute what little we may to the progress of physiology; and, if we succeed in any degree in the attainment of this object, we care not if we should sometimes be found wanting in our capacity of critics: nay, though our articles should occasionally be censured as having little or no reference to the works with whose titles they are associated.

We have so frequently adverted to the leading topics of special physiology, that we shall not at present enter on such themes as respiration, animal heat, &c., on which all has been said that can be said, till new facts afford new materials for discussion. In this article we shall direct our observations where they seem most likely to be useful, without any reference to the connexion of the subjects.

### *Characteristics of Man.*

These are *mental* and *corporeal:* on the former, Dr. Elliotson makes the following remarks.

"In judging of the mental faculties of mankind, not merely those should be considered which an unfortunately situated individual may display, but those which all the race would display under favourable circumstances. A seed and a pebble may not on a shelf appear very dissimilar, but, if both are placed in the earth, the innate characteristic energies of the seed soon became conspicuous. A savage may in the same manner seem little superior to an orang utan, but, if instruction is afforded to both, the former will gradually develope the powers of our nature in their noble superiority, while the latter will still remain an orang utan. The excellence of man's mind demonstrates itself chiefly by his voice and hands. Witness the infinite variety and the depth of thought expressed by means of words: witness his great reasoning powers, his ingenuity, his taste, his upright, religious, and benevolent, feelings, in his manufactories, his galleries of the fine arts, his halls of justice, his temples, and his charitable establishments. Besides the qualities common to all animals, each of which he, like every animal, possesses in a degree peculiar to himself, and some indeed in a degree very far surpassing that in which any brute possesses them, for instance, benevolence, mechanical contrivance, the sense for music and language, and the general power of observation and inference respecting present circumstances, he appears exclusively gifted with at least feelings of religion and justice, with taste, with wit, and with decided reflecting faculties of comparing and reasoning into causes." (P. 7.)

It is observed by Galen, that, the body being the organ of the mind, the corporeal structure of every animal is adapted to its particular dispositions and faculties.* This is true; but it is no less so, that the mind is in a great degree dependent for its development on the peculiarities of bodily conformation. While it is probable, as Locke has taught, that we originally derive all the materials of thought from the external world, through the medium of the senses, it is still more evident that the very organs which we use to express our thoughts and emotions become the means of increasing indefinitely the number of our ideas. The principal organs of this kind are those of speech and the hand. There is no doubt that "the excellence of man's mind demonstrates itself chiefly by his voice and hands;" but is it not from the possession of these singular endowments that much of his mental excellence is derived? This subject has not been sufficiently attended to by naturalists. The physiology of speech, and the philosophy of language, have yielded materials for much interesting disquisition: the mechanism of the hand supplied Galen† with the subject of the most masterly anatomical description that ancient or modern times have produced, and has recently been placed in a beautiful relation to natural theology by one of the most accomplished writers of the present day; but the *united* influence of speech and the hand, on the intellectual and moral development of man, has not generally been sufficiently recognized.

Language is the great medium through which knowledge

* De Usu Partium, lib. i. † Idem.

is first communicated, but the hand renders it cumulative and transmissible from one generation to another. In how many ways does this wonderful organ embody and multiply the products of thought! It gives permanency to the record of the historian, to the song of the poet, and the precepts of the sage. It reveals the fine conceptions of the painter and the sculptor, which, but for its aid, must have remained for ever hidden in the depths of the creative mind, and sends them forth, to generate in other minds new forms of beauty. Without the hand, in short, knowledge could only have been obscure and traditionary: all the imitative arts must have been rude, and several of them could have had no existence: many of the chief sources of thought would have remained unopened, and, if man had ever attained to civilization, it must have been by a process inconceivably slow and toilsome. For a brief but comprehensive and original view of the advantages which man derives from the cooperation of the hand with the power of articulate speech, we beg to refer the reader to Dr. Haslam's work on "Sound Mind;" a work no less remarkable for vigour of thought than for elegance of style, but which, we fear, is less familiar to the profession than it deserves to be.

In comparing the mental faculties of man and the lower animals, much confusion has arisen from the undefined use of the terms *reason* and *instinct*, and from opposing them to each other, as if instinct were to animals what reason is to man. The truth is, the two powers are entirely distinct in *kind*, and both exist in man as well as in animals, although in the former reason, and in the latter instinct, predominates. Dr. Fleming is the only author with whom we are acquainted who has treated the subject of instinct philosophically, and shewn satisfactorily wherein the instincts of man differ from his intellectual powers.

"The impressions," says this author, "which are made upon us by external objects, or the ideas of reflection suggested by memory, when they are the subjects of our intellectual powers, do not necessarily lead to any control over the body in consequence of an act of volition. Between impression and action, there is always a process of thinking, varying greatly in its nature and duration, according to the subject, but absolutely necessary to connect the one with the other.

"In the powers which we are now to consider, the case is very different. Here action follows impression *immediately*. There is no thinking,—no deliberation. There is likewise a difference in the nature of the action in the two cases. There is an *effort* required to perform that which is the result of the intellectual process, whereas, the action which follows in reference to our instinctive powers, is *spontaneous,* or rather, it requires an effort to resist obedience to the impulse.

"As the impressions, in the case of the intellectual powers, are variously modified by the thinking process, the corresponding actions exhibit, in their character, a great degree of variety. The impressions in the case of the instinctive powers, suffering no intermediate modifications, produce actions characterized by great uniformity.

"In many cases, when an impression is produced upon us, which, as belonging to the instinctive powers, would have been followed by action, the intellectual powers interpose their control, and the impression is surveyed before action is permitted. But, as might have been expected, such action is less varied, than when originally the result of an intellectual process, but more irregular than in those cases in which impression and action follow instantaneously. In the one case, the action is modified by the impression; in the other, by the changes the impression has undergone by thought.

"The powers to which we are now directing our attention, are usually denominated, by the writers on the science of mind, ACTIVE POWERS. To this appellation, however, there are strong objections. There are other powers which excite to action, inseparably connected with our constitution, which do not belong to this class. Some actions are produced by irritability, of which we are not conscious. Action is likewise produced by the information obtained by the senses, through the medium of thought, or in consequence of the ideas of reflection which spontaneously arise in the mind. Hence the difference between the intellectual and instinctive powers, is not so distinctly marked in the acts of volition as in the manner in which these acts are excited." (*Philosophy of Zoology*, vol. i. p. 241.)

The consideration of the human instincts opens an extensive, but hitherto little cultivated, field of inquiry. The study of their natural influence on our physical and moral constitution, and of the effects arising from their morbid exaltation, deficiency, or perversion, has an important relation to all those investigations wherein we have to regard man in a double light,—as a material organism, and as an intellectual and moral being: hence its bearing on the subject of insanity, to which we ventured to allude in our late review of Dr. Prichard's work.

As the result of his superior reasoning faculties, man is the only animal capable of religion. Dr. Elliotson justly observes, that the power of investigating causes belongs exclusively to man, and on this depends the recognition of a *first cause,* which is the basis of all religion. The notion that religion is innate, and the idea of a Deity impressed on the human mind by the Creator, is completely overturned by the reflection that, if God had impressed on the minds of his creatures an idea of himself, it would certainly have been a correct one: whereas, we know well that any thing like a just conception of the Deity among mankind has resulted only from the light of divine revelation, or from the deductions of reasoning in a very advanced period of philosophy. (Vide *Locke on Human Understanding*, vol. i. p. 57.)

The following account of the *corporeal* characteristics of man is so correct and comprehensive, that we quote it entire, without comment or addition.

"The *corporeal* characteristics of mankind are not less striking and noble. Among the beings beheld by Satan in Milton's Paradise,

> "Two of far nobler shape, erect and tall,
> Godlike erect, with native honour clad,
> In naked majesty seem'd lords of all."

"The erect posture is natural and peculiar to man. All nations walk erect, and, among those individuals who have been discovered in a wild and solitary state, there is no well authenticated instance of one whose progression was on all-fours. If we attempt this mode of progression, we move either on the knees or the points of the toes, throwing the legs obliquely back to a considerable distance; we find ourselves insecure and uneasy; our eyes, instead of looking forwards, are directed to the ground; and the openings of the nostrils are no longer the lower part of the nose, in a situation to receive ascending odorous particles, but lie behind it. Our inferior extremities, being of much greater length, in proportion to the others and to the trunk, than the posterior of brutes with four extremities, even in children in whom the proportion is less, are evidently not intended to coincide with them in movement; they are much stronger than the arms, obviously for the purpose of great support: the presence of calves, which are found in man alone, shows that the legs are to support and move the whole machine; the thigh bones are in the same line with the trunk, in quadrupeds they form an angle, frequently an acute one; the bones of the tarsus become hard and perfect sooner than those of the carpus, because strength of leg is required for standing and walking sooner than strength of arm and hand for labour; the great toe is of the highest importance to the erect posture, and bestowed exclusively on mankind; the os calcis is very large, particularly at its posterior projection, for the insertion of the strong muscles of the calf, and lies at right angles with the leg; we alone can rest fully upon it, and in fact upon the whole of the tarsus, metatarsus, and toes. The superior extremities do not lie under the trunk as they would if destined for its support, but on its sides, capable of motion in every direction towards objects; the fore-arm extends itself outwards, not forwards, as in quadrupeds, where it is an organ of progression; the hand is fixed not at right angles with the arm, as an instrument of support but in the same line, and cannot be extended to a right angle without painfully stretching the flexor tendons; the superior extremity is calculated in the erect posture for seizing and handling objects, by the freedom of its motions, by the great length of the fingers above that of the toes, and by the existence of the thumb, which, standing at a distance from the fingers and bending towards them, acts as an opponent, while the great toe is, like the rest, too short for apprehension, stands in the same line with them, and moves in the same direction: were our hands employed in the horizontal posture, they would be lost to us as grand instruments in the exercise of our mental superiority. Quadrupeds have a strong ligament at the back of the neck to sustain the head; in us there is no such thing, and our extensor muscles at the back of the neck are comparatively very weak. They have the thorax deep and narrow, that the anterior extremities may lie near together and give more support; the sternum too is longer, and the ribs extend considerably towards the pelvis to maintain the incumbent viscera; our thorax is broad from side to side, that the arms being thrown to a distance may have greater extent of motion, and shallow from the sternum to the spine; and the abdominal viscera, pressing towards the pelvis rather than towards the surface of the abdomen in the erect attitude, do not here require an osseous support. The pelvis is beautifully adapted in us for supporting the bowels in the erect posture; it is extremely expanded, and the sacrum and os coccygis bend forwards below: in brutes it does not merit the name of pelvis; for, not having to support the abdominal contents, it is narrow, and the sacrum inclines but little to the pubes. The nates, besides extending the pelvis upon the thigh bones in the erect state of standing or walking, allow us to rest while awake in the sitting posture, in which, the head and trunk being still erect, our organs of sense have their proper direction equally as in walking or standing; were we compelled to lie down like quadrupeds, when resting during the waking state, the different organs of the face must change their present situation to retain their present utility, no less than if we were compelled to adopt the horizontal progression; and, conversely, were their situation so changed, the provision for the sitting posture would be comparatively useless.

"While some, perversely desirous of degrading their race, have attempted to remove a splendid distinction by asserting that we are constructed for all-fours, others with equal perverseness and ignorance have asserted that monkeys are destined for the upright posture. The monkey tribe, it is true, maintain the erect posture less awkwardly than other brutes with four extremities, but they cannot maintain it long, and, while in it, they bend their knees and body; they are insecure and tottering, and glad to rest upon a stick; their feet, too, instead of being spread for support, are coiled up as if to grasp something. In fact their structure proves them to be neither biped nor quadruped, but four-handed, animals. They live naturally in trees, and are furnished with four hands for grasping the branches and gathering their food. Of their four hands the posterior are even the more perfect, and are in no instance destitute of a thumb, although, like the thumbs of all the quadrumana, so insignificant as to have been termed by Eustachius, 'omnino ridiculus;' whereas the anterior hands of one variety (*simia paniscus*) have not this organ. The whole length of the orang utan, it may be mentioned, falls very much short of ours.

"It was anciently supposed that man, because gifted with the highest mental endowments, possessed the largest of all brains. But as elephants and whales surpass him in this respect, and the sagacious monkey and dog have smaller brains than the comparatively stupid ass, ox, and hog, the opinion was relinquished by the moderns, and man was said only to have the largest brain in proportion to the size of his body. But as more extensive observation proved canary and other birds, and some varieties of the monkey tribe, to have larger brains than man in proportion to the body, and several mammalia to equal him in this particular, and as rats and mice too surpass the dog, the horse, and the elephant, in the comparative bulk of their brains, this opinion also gave way, in its turn, to that of Sömmerring, that man possesses the largest brain in comparison with the nerves arising from it. This has not yet been contradicted, although the comparative size of the brain to the nerves originating from it (granting that they originate from it) is not an accurate measure of the faculties, because the seal has in proportion to its nerves a larger brain than the house-dog, and the porpoise than the orang utan.

"As the human brain is of such great comparative magnitude, the cranium is necessarily very large and bears a greater proportion to the face than in any other animal. In an European the vertical section of the cranium is almost four times larger than that of the face (not including the lower jaw); in the monkey it is little more than double; in most feræ, nearly equal; in the glires, solipedes, pecora, and belluæ, less. The faculties, however. do not depend upon this proportion, because men of great genius, as Leo, Montaigne, Leibnitz, Haller, and Mirabeau, had very large faces, and the sloth and seal have faces larger than the stag, horse, and ox, in proportion to the brain, and the proportion is acknowledged by Cuvier to be not at all applicable to birds. We are assisted in discovering the proportion between the cranium and face by the facial angle of Camper. He draws two straight lines, the one, horizontal, passing through the external meatus auditorius and the bottom of the nostrils; the other, more perpendicular, running from the convexity of the forehead to the most prominent part of the upper jaw. The angle which the latter, the proper facial line, makes with the former, is greatest in the human subject, from the comparative smallness of the brain and the great development of the mouth and nose in brutes. In the human adult this angle is about from 65° to 85°; in the orang utan about from 55° to 65°; in some quadrupeds 20°; and in the lower classes of vertebral animals it entirely disappears.

"Neither is it to be regarded as an exact measure of the understanding, for persons of great intellect may have a prominent mouth; it shows merely the projection of the forehead, while the cranium and brain may vary greatly in the size of other parts; three fourths of quadrupeds, whose crania differ extremely in other respects, have the same facial angle; great amplitude of the frontal sinuses, as in the owl and hog, without any increase of brain, may increase it. and for this reason Cuvier draws the facial line from the internal table of the frontal bone.

"In proportion as the face is elongated, the occipital foramen lies more posteriorly; in man consequently it is most forward. While in man it is nearly in the centre of the base of the cranium, and horizontal, and has even sometimes its anterior margin elevated; in most quadrupeds it is situated at the extremity of the cranium obliquely, with its posterior parts turned upwards, and is in some completely vertical. On this difference of situation, Daubenton founded his occipital angle. He drew one line from the posterior edge of the foramen to the lower edge of the orbit, and another, in the direction of the foramen, passing between the condyles and intersecting the former. According to the angle formed, he established the similarity and diversity of crania. The information derived from it in this respect is very imperfect, because it shows the difference of the occiput merely. Blumenbach remarks that its variations are included between 80° and 90° in most quadrupeds which differ very essentially in other points.

"The want of the ossa intermaxillaria has been thought peculiar to mankind. Quadrupeds, and nearly all the ape tribe, have two bones between the superior maxillary, containing the dentes incisores when these are present, and termed ossa intermaxillaria, incisoria, or labialia. But these do not exist universally in them. Man only has a prominent chin: his lower jaw is the shortest, compared with the cranium, and its condyles differ in form, direction, and articulation, from those of any brute: in no brute are the teeth arranged in such a close and uniform series; the lower incisores, like the jaw in which they are fixed, are perpendicular, a distinct characteristic of man, for in brutes they slope backwards with the jaw bone; the canine are not longer than the rest, nor insulated as in monkeys; the molares differ from those of the orang utan and of all the genus simia by their singularly obtuse projections.

"The slight hairiness of the human skin in general, although certain parts, as the pubes and axillæ, are more copiously furnished with hair than in brutes; the omnivorous structure of the alimentary canal; the curve of the vagina corresponding with the curve of the sacrum formerly mentioned, preventing woman from being, as brute females are, retromingent; the peculiar structure of the human uterus and placenta; the length of the umbilical chord and the existence of the vesicula umbilicalis until the fourth month; together with the extreme delicacy of the cellular membrane; are likewise structural peculiarities of the human race. The situation of the heart lying not upon the sternum, as in quadrupeds, but upon the diaphragm, on account of our erect position, the basis turned not, as in them, to the spine, but to the head, and the apex to the left nipple; the absence of the allantois, of the panniculus carnosus, of the rete mirabile arteriosum, of the suspensorius oculi; and the smallness of the foramen incisivum, which is not only very large in brutes, but generally double, though not peculiarities, are striking circumstances.

"Man only can live in every climate; he is the slowest in arriving at maturity, and, in proportion to his size, he lives the longest of all mammalia; he only procreates at every season, and, while in celibacy, experiences nocturnal emissions. None but the human female menstruates.

"Man, thus distinguished from all other terrestrial beings, evidently constitutes a separate species. For 'a species comprehends all the individuals which descend from each other, as from a common parent, and those which resemble them as much as they do each other;' and no brute bears such a resemblance to man." (F. 8.)

*Mind.* In the third chapter, containing a "general view of the organs, functions, and powers of the human body," our author introduces some remarks on mind, which are not characterized by his usual good sense, and in which he is manifestly led astray by *phrenology,* the pseudo-science, of which he is a well-known supporter. As we shall probably have occasion, in reviewing the second part of Dr. Elliotson's work, to speak at some length on the subject of phrenology, it may not be out of place here to offer some reasons for dissenting from the too-prevalent materialism of the day, which is usually, though not necessarily, connected with that fantastic hypothesis.

The following is a sample of our author's philosophy of the mind.

"The ANIMAL FUNCTIONS demonstrate MIND. This is seated in the brain, to which the spinal marrow, nerves, and voluntary muscles, are subservient. MIND is the functional power of the living brain. As I cannot conceive *life* any more than the power of attraction unless possessed by matter, so I cannot conceive *mind* unless possessed by a brain, or by some nervous organ, whatever name we may choose to give it, endowed with life. I speak of terrestrial or animal mind; with angelic and divine nature we have nothing to do, and of them we know, in the same respects, nothing. To call the human mind positively a ray of the divinity, (*Divinæ particula auræ, Ex ipso Deo decerptus, Ex universa mente deli-*

*batus*,) appears to me absolute nonsense. Brutes are as really endowed with mind, with a consciousness of personality, with feelings, desires, and will, as man. Every child is conscious that it thinks with its head, and common language designates this part as the seat of mind. Observation shows that superiority of mind in the animal creation is exactly commensurate with superiority of brain; that activity of mind and of brain are coequal; and that, as long as the brain is endowed with life, and remains uninjured, it, like all other organs, can perform its functions, and mind continues; but, as in all other organs, when its life ceases, its power to perform its function ceases, and the mind ceases; when disease or mechanical injury affects it, the mind is affected, inflammation of the stomach causes vomiting, of the brain delirium, a blow upon the loins suppression or alteration of the urine, a blow upon the head stuns; if originally constituted defective, the mind is defective; if fully developed, and properly acted on, the mind is vigorous: accordingly, as it varies with age, in quality and bulk, is the mind also varied, the mind of the child is weak and very excitable, of the adult vigorous and firm, and of the old man weak and dull, exactly like the body; and the character of the mind of an individual agrees with the character of his body, being equally excitable, languid, or torpid, evidently because the brain is of the same character as the rest of the body to which it belongs, the female mind exceeds the male in excitability as much as her body; the qualities of the mind are also hereditary, which they could not be, unless they were, like our other qualities, corporeal conditions; and the mind is often disordered upon the appearance of a bodily complaint, just as other organs, besides the brain, are affected under similar circumstances; the retrocession of an eruption may affect the lungs, causing asthma; the bowels, causing enteritis; or the brain, causing insanity; phthisis and insanity sometimes alternate with each other, just like affections of other organs; the laws of the mind are precisely those of the functions of all other organs, a certain degree of excitement strengthens it; too much exhausts it; physical agents affect it, and some specifically, as is the case with other functions, for example, narcotics. The argument of Bishop Butler, that the soul is immortal and independent of matter, because in fatal diseases the mind often remains vigorous to the last, is perfectly groundless; for any function will remain vigorous to the last, if the organ which performs it is not the seat of the disease, nor much connected by sympathy, or in other modes, with the organ which is the seat of the disease, the stomach often calls regularly for food, and digests it vigorously, while the lungs are almost completely consumed by ulceration. All the cases that are adduced to prove the little dependence of the mind upon the brain, are adduced in opposition to the myriads of others that daily occur in the usual course of nature, and are evidently regarded as extraordinary by those who bring them forward. An exact parallel to each may be found in the affections of every other organ, and each admits of so easy an explanation, that it may be always truly said, 'Exceptio probat regulam.'" (P. 32.)

We have in this passage a direct appeal to consciousness, in proof of the assertion that the brain thinks. "Every child is conscious that it thinks with its head:" *à fortiori,* then, every man is conscious that he thinks with his head. Now, either this is not true, or we must relinquish all title to humanity; since, after the most careful attention, we cannot detect in ourselves any consciousness whatever of thinking with any one part of our body, rather than another, or of thinking with any part of our body at all. We have here a right to meet our author's assertion with a flat negation, without any reasoning about the matter; and we say, peremptorily, that every man is not conscious of thinking with his head; for we are men, but are not conscious of any such thing.

We do not at all mean to impugn Dr. Elliotson's consciousness: for aught we know, his head may lucubrate, and be fully aware of its function; but we can assure the Doctor, that if his head thinks the most difficult point in the philosophy of man is to be settled by a few random assertions, it thinks wrong.

Our author illustrates his views by divers quotations from bishops, philosophers, and wags, whose authority on this subject may be allowed about equal weight; since, as Samuel Johnson truly observes, "on the arena of conjecture all men stand equal, who are equally well informed;" and since, as we shall endeavour to shew, no man living possesses any real information on the matter: the *arguments* of the immaterialist being inconclusive, from the abstract nature of the inquiry; and the *facts* of the materialist being as explicable on one hypothesis as the other.

Conceding, what might very fairly be disputed, that the manifestations of the mind are exactly commensurate with the development of the brain, and uniformly disordered by its lesions, the cause of the materialist gains nothing by the admission; for if the brain be, as few will deny, the medium through which the thinking principle receives all those impressions from which ideas are derived,—the storehouse, as it were, in which the primary materials of thought are laid up, —it follows, of course, that, the more perfectly the brain is organized, the more perfect will be the materials of thought, and the higher the development of the mind; that, when its functions are disordered, the operation of the mind must be deranged; and that, when they cease, the manifestations of the mind, *connected with our present state of existence,* must cease along with them. But it by no means follows that, because thought ceases to be appreciable to us as *corporeal* beings, the thinking principle ceases to exist, or that, because a dead brain no longer supplies the materials of thought, or dead organs of speech the means of communicating it, the disembodied spirit may not find other materials and other means of communication.

In reasoning on the nature of mind, our author evinces a stronger addiction to his own hypothesis than to the principles of logic. "With angelic and divine nature," he tells us, "we have nothing to do:" which is tantamount to saying, that, in considering the hypothesis of materialism, we are to put out of the question the very thing that militates most strongly against it; a very simple way of establishing any position. We admit that we have nothing to do with angelic minds, because we know nothing about them; but the divine mind is, in several of its operations, very distinctly revealed to us in creation. Every man, who uses his understanding, is convinced that the Deity wills and adapts causes to effects, to accomplish his will; and, if we go no further even than this, we may perceive, in the divine mind, operations similar in kind to those of our own: again, no man in his senses believes that the Deity has a brain; hence it is clear that there is at least one mind, and that the most perfect in the universe, whose manifestations are independent of a brain.

If it be asked, what relation has all this to the physiology of the human mind, we answer, that it bears upon it very emphatically, by shewing that *mind* is not in its essence necessarily dependent on organization; and, consequently, that if the facts adduced by the materialist to prove such dependence in the instance of the human mind, be altogether equivocal, and equally reconcileable with an opposite supposition, the balance of reasoning evidently inclines against him. Whoever views this subject candidly and philosophically will, we think, admit that the question cannot be resolved in the present state of our knowledge: at the same time, we do not wish to disguise our conviction that reasoning, as far as it goes, is extremely adverse to the materialist.

Independently of the considerations already urged, there seems little probability that the mental phenomena depend on the brain as their efficient cause, from the absence of all intelligible relation between the cause and the supposed effect. The brain is a material organ; the phenomena of the mind have nothing in common with matter. We defy any man to shew us a single unequivocal instance in which thought, or the smallest approximation to it, results from any material agency, however powerful, or however subtle. The powers of inert nature work their wonders in the earth and air: unknown atmospheric causes generate aeroliths, for whose production we can by no means account; and the diamond is formed in its bed, by a process which we can neither understand nor imitate; but was an *idea* ever generated in the air? was a *syllogism* ever discovered in the bowels of the earth?

In living bodies, again, we meet with results still more complex and wonderful than those produced by inanimate nature, but without approximating a step nearer to thought. The blood, for example, is quite different from any thing to be found in inanimate nature, yet it is a strictly material product: we do not know whether it is alive or not; there are many things about it that we do not comprehend; but it is nevertheless perfectly cognizable to our senses, and is no more like a production of the mind than so much pure water. The muscles of the larynx present an inconceivable rapidity and variety of motion, but it is still mere motion; a phenomenon as strictly mechanical as the falling of the rudest stone to the earth. In short, let

> "science search on weary wing,
> By shore and sea, each mute and living thing,"

and she will find only material results from material agencies, in the most complex as in the simplest arrangements of matter, whether organized or inorganic.

When, therefore, we are told that the brain produces results differing not in *degree* but in *kind,*—in their very nature and essence,—from those produced by any other material substance, we require strong proof of such an allegation; and when we find nothing adduced but a series of phenomena, which accord equally well with either of two very opposite hypotheses, we are certainly not inclined to cede the deductions of our reason, though confessedly inconclusive, in favour of a mere opinion, which happens to be at present in fashion; nor can we sufficiently deprecate the unphilosophical manner in which Dr. Elliotson dogmatises on a subject, of which

> "All that we know is, nothing can be known."

### *Different Stages of Development in the Animal Body.*

This is a topic of considerable interest in general physiology, but which till lately has entirely escaped attention, from the want of due discrimination between simple growth and development. It is not alluded to by our author, nor treated of by any systematic writer on physiology; and for that very reason we introduce it here. The scientific world is indebted to M. Isidore G. St. Hilaire, for tracing an outline of the subject, which it is to be hoped he may find materials for filling up. We make no apology for the following translation from his work on the Anomalies of Organization.

"In that series of phenomena, hitherto so little observed, but in reality so worthy of attention, which, in a certain number of years, mark the transition from infancy to youth, and from this to adult age, we should carefully distinguish those connected with simple *growth* of the frame, from those which characterize its *development*. Growth results from the gradual augmentation of all parts of the body, quite independently of their number, structure, or functions: development, on the contrary, consists essentially in a modification,—a change more or less obvious. In simple growth, all the previous conditions of organs are preserved, except their volume: every true development, on the contrary, may be compared to the metamorphoses of the fœtus, though the changes be less extensive and remarkable. The first appearance of the deciduous teeth,—that of the permanent teeth,—and the period of puberty, are the three principal epochs of development in man, and the animals most nearly allied to him. After each of these, the general growth is usually more or less retarded. Thus, the growth is extremely rapid till the appearance of the first set of teeth; somewhat less so from this time till the appearance of the second set; and still a little less till the period of puberty; after which it undergoes a remarkable diminution. When, indeed, the individual has attained complete puberty,—that is to say, perfect aptitude for the generative functions,—the increase of stature becomes almost insensible, or is entirely arrested.

"Growth and development, although essentially distinct, have nevertheless a very intimate relation to each other. A certain degree of growth of the whole frame induces a new development, and every development, in its turn, indicates the termination of one period of growth, and the commencement of another, in which the growth proceeds more slowly; as if the apparatus newly developed had appropriated, and concentrated in itself, a great part of the formative energy, which was before more equally distributed among all the organs; or, (in more exact terms, and banishing the abstract phraseology too long used by anatomists,) because the apparatus newly developed necessarily diminishes the activity of the general nutrition, by the increased activity of its own.

"These observations apply universally, as well to the earlier periods of development as to that of puberty. If an infant is remarkable for the rapidity of its growth, and surpasses the ordinary dimensions at the age of two, three, or four months, the teeth almost always speedily appear; and it has even been sometimes observed, that, in very large children, the development of the teeth has preceded birth, or followed it almost immediately. On the other hand, where the general growth of an infant is unusually slow, there is almost always a corresponding retardation of the dentition.

"The same remarks hold good with respect to the second dentition as the first; and a comparison of the different species of animals most nearly allied to man in their organization, discovers the same constant relation between the rapidity of the general growth and the protrusion of the deciduous and permanent teeth. But it is especially between puberty and the general growth that an intimate and very remarkable relation subsists. It may be stated, in a general way, that very precocious growth induces also premature puberty, while complete puberty, in its turn, puts a stop to the general growth. Thus we find, as it were, a circle formed by a series of remarkable phenomena, of which one, resulting from that which precedes it, reacts upon the latter, and causes its cessation." (*Histoire, génèrale et particulière, des Anomalies de l'Organisation*, tom. 1er. p. 188.)

These views are very felicitously applied by M. St. Hilaire to the explanation of dwarfishness and gigantic stature: they are, however, no less important in their bearing upon other physiological subjects.

Even in the vegetable kingdom, facts are not wanting illustrative of the relation of growth to development. Thus, the development of the flowers in plants may, in some sort, be considered as their *puberty;* and the very rapid growth of annuals previous to this development, and the cessation of growth which follows it, present an interesting parallel with the phenomena of the corresponding period in animals. The developments which occur in animals and vegetables constitute one of the most remarkable distinctions between organised and inorganic bodies.

"All animals and vegetables," says Professor Tiedemann, "as soon as they proceed from the shoot, from the reproductive corpuscule, from the ovum or the seed, appear in a simple form, which they gradually leave, to take a more complicated one; this is accompanied by an increase in the diversity and intensity of their manifestations of activity. All organic bodies, whose life is not curtailed or interrupted in any way, exhibit three distinct periods in their existence, that of gradual growth, or youth, of complete development, or sexual maturity, and that of decay, or old age, which finishes in death. In this manner, the representatives of all the vegetable and animal species are included in a continual series of changes, the duration of which varies infinitely in the different groups of living bodies, being extended from the space of a few days to entire ages. To be born, to be developed, to engender, and to die, are acts occurring uninterruptedly in the domain of organic bodies, first calling into existence, then repelling into nothingness the individuals representing the species. These changes, which happen only once in each individual, are dependent on the mode of action of the organic powers, whose activity commences in its minimum of intensity, by degrees arrives at the maximum, is exhausted by the very fact of its exercise, and ends by being extinguished.

"In inorganic bodies no changes occur that can be compared to those of the periods of life or the ages. Substances once united into crystals, according to the laws of attraction, and whose molecules are held together by cohesion, remain in their respective forms, without our perceiving in them thenceforward any phenomena presenting even a distant analogy to those of development and the ages of living bodies. Crystals of themselves undergo no changes that terminate in their ruin and destruction in a certain lapse of time. If they last a few hours only, or thousand of years, it is dependent altogether on accidental circumstances, according as they enter into connexion with matters which have a stronger affinity for the substances that constitute them." (*Tiedemann's Physiology, translated by* Drs. Gully and Lane, p. 45.)

The different kinds and periods of development have not hitherto been regarded in due connexion with each other, and with the general growth; but an intelligent inquirer, keeping in view the theory of St. Hilaire, and pursuing it through the whole range of comparative physiology, might possibly succeed in establishing some important generalizations.

But it is time to wind up this article; and we are happy to be able to do so with a high commendation of the work whose title stands at the head of it. We can conscientiously recommend Dr. Elliotson's Physiology as an accurate and comprehensive digest of the science, and an excellent guide to the student on all subjects that have nothing to do with the philosophy of the mind. On this alone, notwithstanding our author's *materialism*, we would strongly advise the young physiologist to regard all that he says as quite *immaterial.*

